# Influence of the relative age effect and maturity status on competitive playing time in an elite football academy

**DOI:** 10.3389/fspor.2026.1815789

**Published:** 2026-06-01

**Authors:** Ignacio Díaz-Almazán, Diego Muriarte, Manuel Barba-Ruiz, Francisco Gallardo Mármol, Rodrigo García de la Chica, Adrián Martín-Castellanos

**Affiliations:** 1 Departamento de Deportes, Facultad de Ciencias de la Actividad Física y del Deporte (INEF), Universidad Politécnica de Madrid, Madrid, España; 2 Facultad de Ciencias Biomédicas y de la Salud, Universidad Alfonso X El Sabio, Villanueva de la Cañada, Madrid, Spain

**Keywords:** birth quartile, maturity status, relative age effects, talent selection, youth soccer

## Abstract

**Introduction:**

The Relative Age Effect (RAE) could influence talent identification and playing opportunities in youth football. The aim of this study was to examine the influence of RAE and maturity status on competitive playing time in an elite football academy.

**Methods:**

A cross-sectional study was conducted looking at 172 cases of male youth football players (U12-U15). Although data were collected over two seasons (2023-2024 and 2024-2025), the analyses did not include between-season comparisons. Accumulated competitive minutes played across the season were analyzed using a linear mixed-effects model, and associations between the categorical variables were examined using the chi-square test.

**Results:**

The findings showed that none of the primary effects were statistically significant. The results suggest a trend in the expected direction; however, the effect was not statistically significant for status, (*p* = .057), and quartile, (*p* =.054). Late-maturing players were associated with a significant decrease in playing time (minutes) (*p* = .017). In contrast, being a defender (*p* = .035), and belonging to quartile 2 (*p* = .034) were linked to significant increases in playing time (minutes). There were more midfielders with late development (*adjusted residual* = 3.7), and more defenders with early development than expected by chance (*adjusted residual* = 1.98).

**Discussion:**

These findings suggest that both RAE and maturity status influence playing opportunities in elite youth football, highlighting the need for development-oriented competition structures.

## 1 Introduction

Within sports activities, grouping by chronological age is a common tool for establishing different competition categories. The annual calendar begins on January 1, and this chronological date is used to categorize players into specific age groups for training and competition (1), more specifically, in football, the competitive season typically spans from August to May. The competition is divided into categories according to two years of age, since the difference between the players in each category can be as much as 24 months (2). Players born earlier in the competitive selection period could be potentially more developed psychologically, physically, and cognitively than their teammates (3, 4). This could give older players a competitive advantage (5) compared to those born relatively later in the year, which may be reflected in the age differences among those selected in the same year (6). This disparity may confer potential physical and psychological advantages to players over their young peers, increasing their likelihood of selection and contributing to what is known as the relative age effect (RAE) (7). This effect is commonly observed in team sports, particularly in those that emphasize physical strength, such as football (8–11). The development of physical performance abilities remains a key component in talent identification. However, other aspects of performance such as technical, tactical, psychological and social factors, are also essential criteria for accurately assessing a playeŕs quality and long-term potential, and they can further support both talent identification and development (12, 13) Therefore, it could be said that the determining factors of the RAE are multifactorial (14–16). The effect of the RAE during adolescence is attributed to increased selection and playing opportunities for players born in the early months of the year (17). Additionally, there are consistent findings regarding the prevalence of players born at the beginning of the year. Studies have shown that RAE tends to benefit those born at the beginning of the year (18, 19). In a study of 1,212 male players aged 8–18 years from 17 professional football academies in the UK, those born in the first quartile accounted for 49% of selected players, while only 9% were born in the fourth quartile (20). This mechanism of RAE is relevant for the coaches of early-stage performance teams seeking the latest results (21). It is also worth noting the work carried out by Helsen et al. (22), who reported that football players born in the last months of the year were more likely to drop out of the sport at an early age, which is why players born in the last months must work harder and obtain more opportunities to develop their football careers.

Although the RAE is well documented across a range of countries and age groups, few studies have examined its impact on playing time in youth players aged U12–U15 in elite clubs or how this effect persists over time (23). European top-level soccer clubs continually seek out the most talented players, identifying them at increasingly younger ages (24). There is limited research that examined whether players' relative age affects their playing time in competition, and the findings have been inconclusive (25–27). Playing time in competition is a key factor in the development of young talent. Many players are willing to change clubs in order to gain more minutes on the field (26). The results obtained in a professional football academy in Spain showed that players born in the first quartile did not play more minutes in competition than those born during the rest of the year (28). Building on this existing evidence, the present study aims to investigate the relationship between RAE, maturity status and playing time in youth football players (U12–U15), particularly within elite academy contexts.

When considering the selection, evaluation, and performance processes of young team sport athletes, relative age and maturation status are key factors to take into account. Specifically, biological maturation is defined by timing of progression toward a mature state, whereas relative age refers to the chronological age differences among individuals born in the same year, which may result in physical and psychological differences between early-, on time-, and late- maturing players within the same calendar year (29). Maturity status in team sports, such as football, also plays an important role in influencing both players selection processes and shaping their development process in sports (30). The consequence of greater maturational development can lead to increased selection and greater participation in competition compared to younger players (31). Specifically, high-performance youth academies aim to develop the talent of young players focused on professional soccer (32), where they have an academy infrastructure dedicated to identifying and developing talent in young players (33). Previous studies have indicated that RAE and biological maturity may interact in youth football, particularly in elite settings, where players born earlier in the selection year are more likely to exhibit advances maturity status, potentially influencing selection and performance outcomes (34, 35).

The objective of this study is to perform a descriptive analysis of the influence of the relative age effect and maturity status on playing time in competition across four youth football categories over two seasons.

## 2 Material and methods

### 2.1 Participants

A cross-sectional study design was used. The research was conducted with the competition teams of a professional youth academy in the Community of Madrid, from 12 to 15 years old, belonging from Under 12 (U-12) to Under 15 (U-15). At the beginning of the season, legal parents sign an informed consent form regarding the processing of players' data, facilitating access to and use of such data. The first team competes in La Liga, in the second and first divisions during those seasons, 2023/2024 and 2024/2025. The total sample included 172 records, corresponding to 136 players. Of these, 36 players participated in both seasons and were therefore included twice, as each season was treated as an independent observation based on their corresponding age group. This research was conducted in accordance with the Declaration of Helsinki and approved by the local ethics committee. (Ref. number FDRED00000-DML-DATOS-20230609).

### 2.2 Variables

The main variable was the birth quartile category, established according to the month of birth relative to the competitive year (selection year cut-off): Q1; January–march, Q2; April–June, Q3; July–September and Q4; October–December. The year of birth was also considered. Another relevant variable was the playeŕs maturity status, which could be categorized as early, on time or late. Playing position was categorized according to the proposal by Peña-González (36): Goalkeepers, defenders, midfielders and forwards. The competitive level was defined according to the club structure: U-12 (Alevín Superliga), U-13 (Infantil División de Honor), U-14 (Infantil Superliga) and U-15 (Cadete División de Honor).

### 2.3 Procedure

Data collection was carried out using different instruments. First, to assess the age of each player, they were given a questionnaire at the beginning of the season in which they had to indicate their date of birth, from which the corresponding birth quartile could be obtained, as well as the position they usually played on the field, assigned by their respective coaches. To analyze the player's maturity status, maturation tests were performed using the SONIC BAUSport instrument (SonicBone Medical, Rishon Le Zion, Israel), which is validated for this type of testing (37–39). This mobile ultrasound estimates the player's skeletal age by measuring bone density at three points on the left hand: wrist, metacarpal and third phalanx, which indicates the athletés maturation status, classifying them into different statuses: players were classified as early-, on time-, or late-maturing based on their estimated age at peak height velocity (PHV), calculated using the maturity offset method proposed by Mirwald et al. (40). Players who reached PHV more than one year earlier than the group mean were classified as early, those within ±1 year of the mean as on time, and those who reached PHV more than one year later than the mean as late (41). This data collection was carried out at the beginning of the season, during the first week of the pre-season for all categories in both seasons. Data from both seasons were included in the analysis and treated as separate observation for each player. To account for the non-independence of repeated measures across seasons, player ID was included as a random effect in the mixed-effects model.

The minutes played in competition were recorded using La Liga's official software, where only official matches were analyzed, excluding friendly matches. All the teams analyzed had 30 official matchdays in competition. The playing time is specific to each category, as established by the competition regulations of the corresponding football federation, which define different match durations according to age group: the duration for the matches of U-12 is 60 min, U-13 and U-14 70 min and U-15, 80 min. To allow comparison across all cases, the percentage of playing time was calculated due to the relevant differences in total minutes among all categories over the 30 matchdays of competition: U-12 (1,800 min), U-13 and U-14 (2,100 min) and U-15 (2,400 min). To ensure data protection, all personal information was anonymized and coded before statistical processing.

### 2.4 Statistical analysis

The analysis was performed using SPSS v24.0 (IBM Corporation, Armonk, NY) and Microsoft Excel v2110, which was used for database management and cleaning. The Chi-square test was used to evaluate the association between birth quartiles and other categorical variables: year of birth, category, and playing position. When significant associations were identified, adjusted residuals were calculated to examine differences between observed and expected frequencies, with values greater than ±1.96 considered statistically significant. Likewise, mixed-effects models were used to account for the non-independence observations arising from repeated measures and hierarchical data structures. These models allow the inclusion of both fixed effects and random effects. All fixed effects were entered simultaneously into the model. Player ID was included as a random effect to account for repeated measures within individuals. Model assumptions were assessed by examining the normality and homoscedasticity of residuals, and model fit was evaluated using the Akaike Information Criterion (AIC) and Bayesian Information Criterion (BIC). This approach provides more accurate estimates and valid inference by properly modeling within-group correlation. Additionally, mixed models are robust to unbalanced data and missing observations. The effect size was also calculated to assess the magnitude of the associations found. The level of significance was set at *p* < 0.05. The graphs were generated using GraphPad Prism version 10.0 (GraphPad Software, San Diego, USA).

## 3 Results

First, it is considered appropriate to provide a descriptive overview of the relationship between playing positions and birth quartiles, as well as between playing positions and maturity status, as presented in Table 1.

**Table 1:** Distribution of players for each season, age groups and playing position.

Season	Group	Players	Goalkeepers	Defenders	Midfielders	Forwards
2023–2024	U-12	21	2	7	5	8
	U-13	25	2	8	10	5
	U-14	19	2	5	7	5
	U-15	21	2	6	6	7
2024–2025	U-12	21	2	8	4	7
	U-13	19	1	7	4	7
	U-14	22	2	8	7	5
	U-15	24	2	7	6	9

Table 2 presents the distribution of players across birth quartiles according to their playing position. This table allows for a detailed examination of how RAE may vary across different positional roles.

**Table 2:** Distribution of players across birth quartiles according to their playing position.

Position	Q1	Q2	Q3	Q4
Goalkeepers	7	5	3	0
Defenders	23	24	6	2
Midfielders	25	13	10	1
Forwards	19	16	16	2

Overall, a higher number of players were concentrated in the earlier birth quartiles (Q1 and Q2) across all playing positions. Midfielders showed the highest number of players in Q1, indicating a stronger relative age effect in this position. Defenders displayed a relatively balances distribution between Q1 and Q2, although with a clear decline in the number of players in Q3 and Q4. Forwards presented a more even distribution across Q1, Q2 and Q3, suggesting a less pronounced relative age effect compared to other positions. In contrast, goalkeepers exhibited an absence of players in Q4, although this may be influenced by the smaller sample size in this position.

Table 3 presents the distribution of players according to maturity status (early, on time and late) across playing positions. This table provides an overview of how biological maturation is distributed within each positional role, allowing for the identification of potential differences in maturation patterns between positions.

**Table 3:** Distribution of players by maturity status and playing position.

Position	Early	On time	Late
Goalkeepers	6	8	1
Defenders	19	31	5
Midfielders	5	30	14
Forwards	13	37	3

The distribution of players across maturity status revealed notable differences between playing positions. Midfielders showed a higher number of late maturity players and a lower number of early maturity players than expected, suggesting a distinct maturation profile in this position. In contrast, defenders presented a higher number of early maturity players, indicating a tendency toward earlier biological development in this role. Goalkeepers and forwards displayed a more balanced distribution across maturation categories, with values closer to those expected. Overall, these findings suggest that maturity status may vary according to playing position, with midfielders and defenders showing the most pronounced differences.

**Figure 1:**
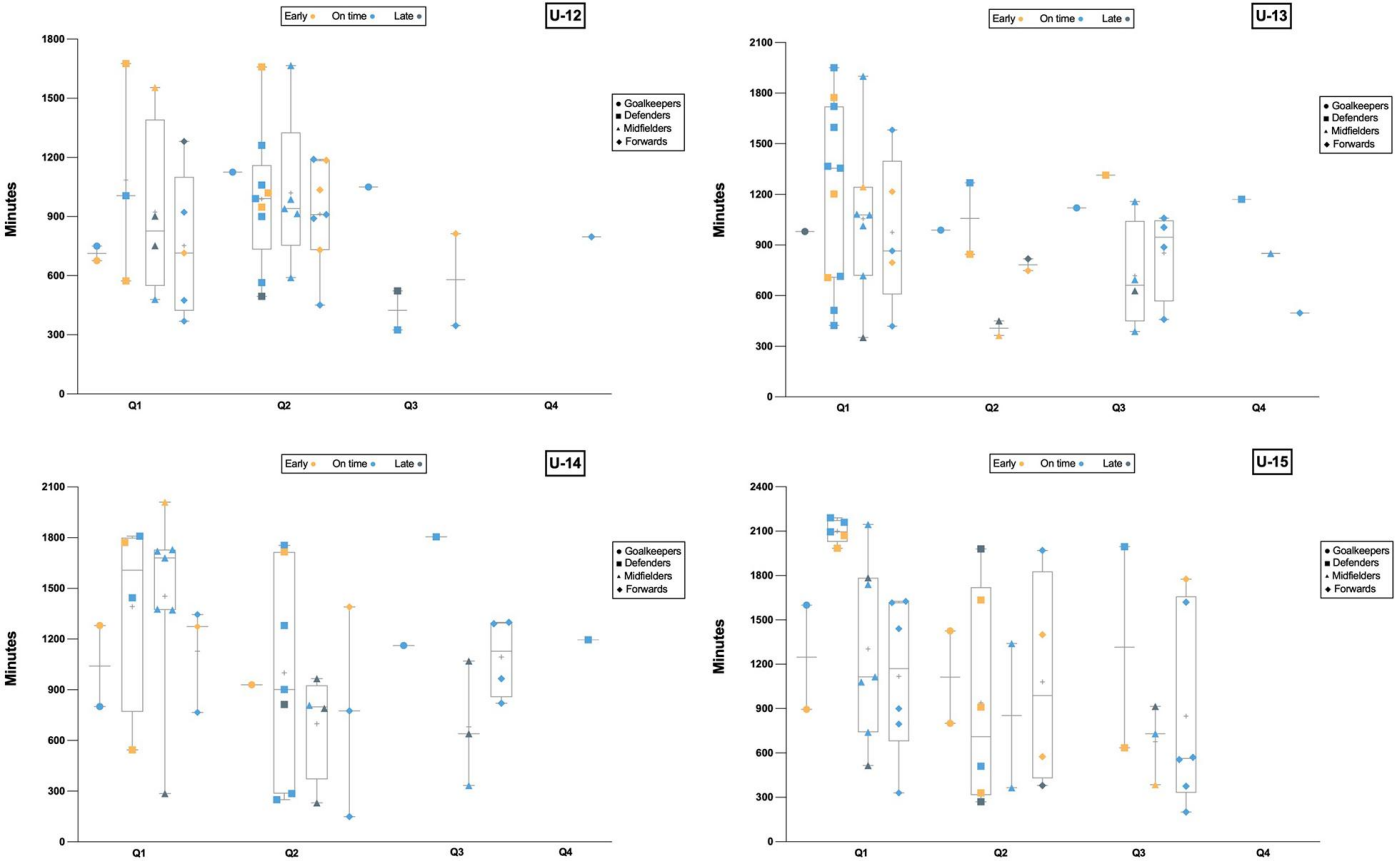
Minutes played in competition according to the category, considering birth quartile, maturity status and position of each particular case.

To provide an overview of the participants related to minutes played, Figure 1 shows the distribution of the total minutes played during the two seasons, considering age group, quartile and position, and stratified by the maturity status.

**Figure 2:**
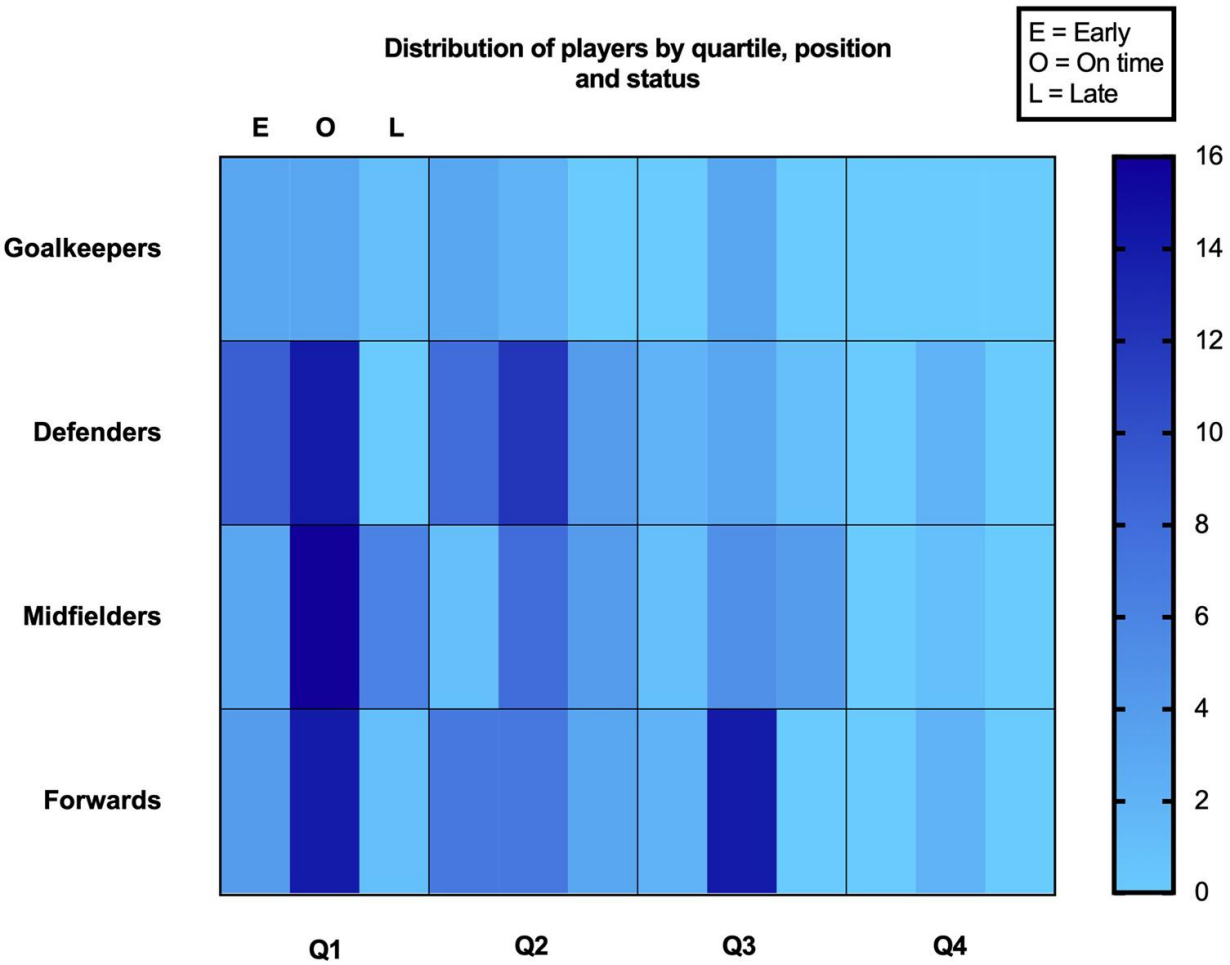
The heatmap shows the distribution of players according to their position, maturity status and birth quartile for the entire sample.

**Figure 3:**
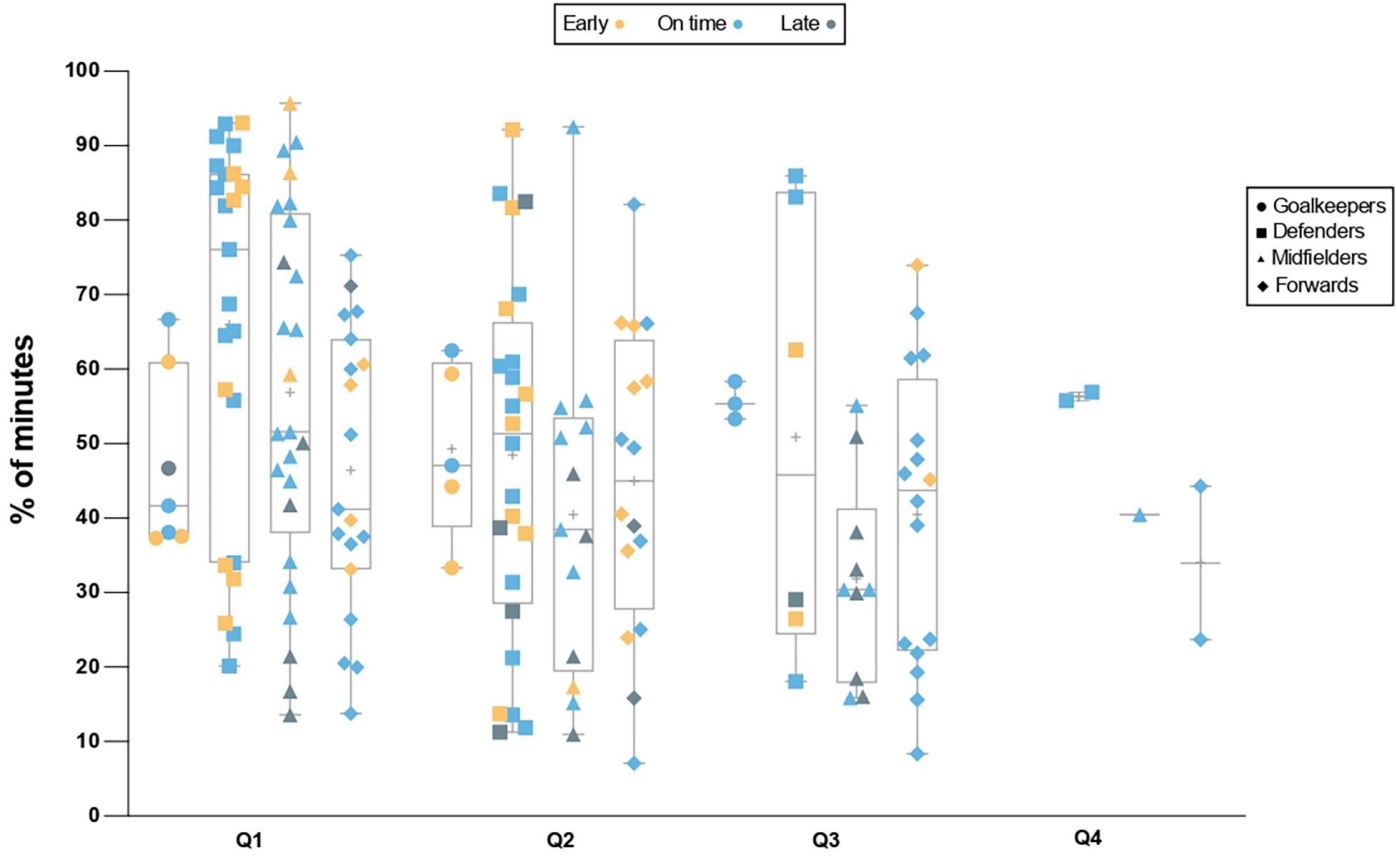
Distribution of the sample according % of minutes played in competition.

A Chi-square test was performed on the data obtained on position and maturity status (*χ²* = 20.72, *p-value* = 0.002, *V* = 0.25). The results suggest a statistically significant association between playing position and maturity status, suggesting that the distribution of maturity status across positions is not random. An overrepresentation of late-maturing midfielders (*adjusted residual* = 3.7), and early-maturing defenders (*adjusted residual* = 1.98), was observed compared to the frequencies expected under the assumption of independence between variables. Expected frequencies under the assumption of independence indicated that midfielders would be distributed as 12.25 early, 30.24 on time and 6.51 late maturity players. Additionally, defenders showed a slightly higher number of early maturity players compared to expected value (13.75). The effect size can be interpreted as small to moderate. However, no association were found between birth quartile and maturity status (*χ*^2^ = 9.03*, p-value* = 0.172, *V* = 0.162), as well as the relationship between birth quartile and position (*χ*^2^ = 9.71, *p-value* = 0.374, *V* = 0.137), suggesting that relative age was not significantly related to biological maturation or positional distribution in this sample. Comparison between observed and expected frequencies under the assumption of independence revealed several deviations across positions and birth quartile. For defenders, a higher number of players than expected was observed in Q2 (24 vs. 18.55), while fewer players than expected were found in Q3 (6 vs. 11.19). Midfielders showed an overrepresentation in Q1 (25 vs. 21.08) and underrepresentation in Q2 (13 vs. 16.52). Similarly, forwards presented a higher number of players than expected in Q3 (16 vs. 10.78) These discrepancies indicate that the distribution of players across quartiles is not entirely uniform across positions, although no consistent pattern was observed across all categories. The descriptive data is reported in Figure 2, which suggests a higher concentration of players in the earlier birth quartiles (Q1–Q2) across most positions. However, no clear or consistent pattern is observed linking birth quartile with maturity status or playing position, supporting the absence of significant associations found in the statistical analysis. Figure 3 presents the distribution of the percentage of minutes played in competition according to birth quartile, maturity status, and playing position.

A linear mixed-effects model was conducted to examine the effects of maturity status, team, position, quartile and seasons on the dependent variable (% of minutes played). Player ID was included as a random effect to account for repeated measures across seasons.

Although no statistically significant effects were found, the results suggest a potential influence of status and quartile on the dependent variable, which may warrant further investigation in future research. The effects of maturity status, *F* (2, 146.10) = 2.918, *p* = .057, and birth quartile, *F* (3, 124.83) = 2.621, *p* = .054, approached but did not reach statistical significance. The effects of category, *F* (3, 89.81) = 1.592, *p* = .197, position, *F* (3, 123.91) = 2.127, *p* = .100, and season, *F* (1,106.35) = 1.854, *p* = .176, were not statistically significant. Model fit was evaluated using information criteria, with an AIC of 1,504 and a BIC of 1,551 reported for the fitted model. These values suggest an acceptable model fit; however, they are primarily informative and are intended for comparison with alternative model.

Fixed effects estimates from the linear mixed-effects model revealed that, relative to the reference categories, (early maturity players, goalkeepers, quartile 1, and U-12) players late matured were associated with a significant decrease in the dependent variable (percentage of minutes played) (*β* = −8.212, *SE* = 3.414, *t* = −2.405, *p* = .017). In contrast, defenders (*β* = 6.639, *SE* = 3.107, *t* = 2.137, *p* = .035) and quartile 2 (*β* = 7.670, *SE* = 3.575, *t* = 2.145, *p* = .034) were associated with significant increases. No statistically significant effects were observed for on time maturity status (*β* = 5.671, *SE* = 2.891*, t* = 1.961, *p* = .052) and U-15 (*β* = 4.403, *SE* = 2.457, *t* = 1.792, *p* = .077).

## 4 Discussion

The aim of this study was to descriptively examine the influence of RAE and biological maturation on playing time in competition across four youth football categories over two seasons. This analysis showed no statistically significant differences in the distribution of either birth quartiles or maturity status among the players included in the study. The main findings of this study indicate that players were predominantly concentrated in the earlier birth quartiles (Q1 and Q2) across all playing positions, reflecting the presence of a relative age effect at a descriptive level. However, this effect did not translate into statistically significant associations between birth quartile and either playing position or maturity status. In contrast, a significant relationship was observed between playing position and biological maturation, with midfielders showing a higher prevalence of late maturity players and defenders a greater representation of early maturity players. Although the mixed-effects model did not reveal statistically significant effects for most variables, maturity status and birth quartile showed values close to significance, suggesting a potential influence on playing time. Notably, late maturity players were associated with a significant reduction in the percentage of minutes played, while defenders and players born in Q2 showed increased playing time compared to the reference categories. Overall, these findings suggest that, while RAE is present in the sample, biological maturation may play a more relevant role in influencing positional distribution and playing time, with the most pronounced differences observed between midfielders (late maturity) and defenders (early maturity).

The overrepresentation of players born in the first two quartiles across most playing positions is consistent wit RAE widely reported in the literature (42–44). In the present study, players born in the earlier months of the year predominated across all positions, although this pattern was less pronounced in forwards. This tendency may be explained by the predisposition to select more physically developed players, which provides greater opportunities for development and performance advantages in competition (45). Indeed, the association between physical development and athletic performance is well established, with relatively older players often demonstrating superior attributes such as height, strength, speed, and agility (46), as well as greater physical output during matches, including higher running speeds and more frequent accelerations (47). In contrast, players born in the later quartiles were more frequently associated with offensive positions, where creative and technical abilities may compensate for physical disadvantages (48). These findings suggest that, although RAE is evident in the overall composition of teams, its influence may vary depending on positional demands. Biological maturation appears to play a key role in shaping positional distribution within the team. In the present study, defenders were predominantly early or on time maturity players, suggesting that this position favors greater physical development. In contrast, midfielders showed a higher proportion of late maturity players and a more heterogeneous maturity profile, which may be explained by the greater technical and tactical demands associated with this role. Forwards and goalkeepers, on the other hand, exhibited a more balanced distribution, with a predominance of on time players. These findings support previous research indicating that physical development influences positional roles, although not all performance related attributes differ significantly across positions. For example, Žuvela and Cikotić (49) found that most motor and functional abilities do not differ significantly between positions, although defenders tend to exhibit greater upper-body explosive strength compared to midfielders. Furthermore, the findings of Khemiri et al. (50) suggest that while physical indicators may contribute to predicting performance, relying exclusively on physical characteristics is insufficient, highlighting the importance of adopting a more comprehensive approach to talent development.

The significant association observed between playing position and maturity status reinforces the influence of biological development on positional allocation. In contrast, no significant associations were found between birth quartile and either maturity status or playing position, suggesting that relative age does not directly determine positional roles or biological development in this sample. These findings partially contrast with previous studies reporting a clear overrepresentation of relatively older athletes within the same category, as observed in other sports contexts (51). However, the lack of a consistent pattern across positions in the present study indicates that the influence of relative age may be context-dependent and moderated by additional factors as tactical roles, competitive level or selection criteria (52, 53). The observed deviations between expected and actual distributions across quartiles further support the idea that, although relative age is present, its impact is not uniform across all positions, suggesting a more complex interaction between physical, tactical, and development factors.

Regarding playing time, previous research has suggested that RAE is not necessarily associated with the number of minutes played in competition, regardless of age (52, 53). However, the results of the present study indicate that players born in the earlier months of the year tend to accumulate more playing time during the analyzed seasons. This may reflect a bias toward selecting players with immediate physical advantages, reinforcing a focus on short-term performance outcomes (54). In addition, late maturity players were associated with reduced playing time, which may limit their immediate competitive opportunities. However, previous research suggests that these players may benefit in the long term, as they often develop greater resilience and adaptability by competing against more physically developed peers (32). Overall, these findings highlight the complexity of talent development processes, suggesting that while physical maturity and relative age may influence short-term opportunities, long-term success may depend on a broader range of factors, including technical, tactical, and psychological development. This reinforces the need for a more holistic approach to player development that goes beyond physical attributes alone.

## 5 Practical applications

In sports, the birth quartile effect requires a review of talent detection and selection systems that assume equal maturity within each group. The differences in performance observed in the formative stages may be due to temporary biological variations rather than greater long-term potential. It is therefore advisable to supplement the assessment of current performance with indicators of future potential and technical-tactical development. Likewise, the adoption of flexible groupings or criteria based on biological maturation can promote more equitable training processes. Training coaches in evolutionary development would help reduce biases associated with relative age. Incorporating age and maturity as contextualizing variables in decision-making would prevent premature discards. Similarly, it is advisable to review early specialization processes when they are conditioned by momentary physical advantages. Taken together, these measures would promote fairer sports development systems geared toward long-term sustainable performance.

## 6 Limitations

This study did not take into account possible injuries to the players analyzed, which could have altered their exposure to competition in some way. Also, in these categories of the club analyzed it is mandatory that all players actively participate in official matches, except for goalkeepers. Nor has the added time in each of the matches analyzed in the sample been taken into account. Due to the small sample size obtained from the last quartile, it is likely that the results obtained for this quartile may be biases when compared with the other quartiles. The existing bias within the team has not been taken into account. Although player ID was included as a random effect in the mixed-effects model, some degree of within-player clustering across seasons may still represent a residual limitation of the study.

## 7 Conclusions

The pursuit of immediate competitive success in high-level youth football appears to prioritize physical development during the formative stages. The findings of this study indicate that a substantial proportion of players were born in the early months of the selection year, supporting the continued presence of the RAE.

This selection bias may grant physically advanced players increased playing time and developmental opportunities, thereby reinforcing cumulative advantages throughout the talent pathway. Consequently, relatively younger or late maturity players may experience reduced competitive exposure, which can contribute to early sport dropout and the underdevelopment of potentially talented individuals. A shift toward a developmental paradigm that prioritizes technical proficiency, tactical understanding, and long-term progression may help mitigate these structural imbalances within youth football.

## Data Availability

The raw data supporting the conclusions of this article will be made available by the authors, without undue reservation.
